# Supplementary material for: Daam2 driven degradation of VHL promotes gliomagenesis
Source: eLife. 2017 Oct 20;6:e31926. doi: 10.7554/eLife.31926 (PMC5650470; doi:10.7554/eLife.31926)
Supplement: Transparent reporting form [file elife-31926-transrepform.docx]

***eLife’s* transparent reporting form**

We encourage authors to provide detailed information *within their submission* to facilitate the interpretation and replication of experiments. If you have any questions, please contact us: [editorial@elifesciences.org](mailto:editorial@elifesciences.org).

**Sample-size estimation**

- You should state whether an appropriate sample size was computed when the study was being designed
- You should state the statistical method of sample size computation and any required assumptions
- If no explicit power analysis was used, you should describe how you decided what sample (replicate) size (number) to use

Please outline where this information can be found within the submission (e.g., page numbers or figure legends), or explain why this information doesn’t apply to your submission:

**Figure 1A.**

- Sample size: We collected molecular data on 10224 tumors of various histological subtypes;

- Refer to 'Method- TCGA database analysis and Comparative Bioinformatics', Line 696-710

**Figure 2G-I.**

- Sample size: 7 Daam2-overexpression and 8 control mice;

- Refer to 'Figure 2. legend (G-I)', Line 1029-1032

**Figure 3C-E.**

- Sample size: 7 mice transplanted with GBM1/Daam2-shRNAi and 5 GBM1/scrambled controls;

- Refer to 'Figure 3. legend (C-E)', Line 1052-1056

**Figure 3F-H.**

- Sample size: 8 Daam2-/- and 9 Daam2+/- mice;

- Refer to 'Figure 3. legend (F-H)', Line 1056-1059

**Figure 5D-G, U.**

- Sample size: 10 Daam2-overexpression, 9 VHL, 6 Daam2+VHL, and 12 control mice;

- Refer to 'Figure 5. legend (D-G, U)', Line 1100-1105

**Figure 2-Supplement 2A-B.**

- Sample size: 17 Daam2-overexpression and 20 control mice;

- Refer to 'Supplemental Figure 2-S2. legend (A-B)', Line 1472-1475

**Replicates**

- You should report how often each experiment was performed
- You should include a definition of biological versus technical replication
- The data obtained should be provided and sufficient information should be provided to indicate the number of independent biological and/or technical replicates
- If you encountered any outliers, you should describe how these were handled
- Criteria for exclusion/inclusion of data should be clearly stated
- High-throughput sequence data should be uploaded before submission, with a private link for reviewers provided (these are available from both GEO and ArrayExpress)

Please outline where this information can be found within the submission (e.g., page numbers or figure legends), or explain why this information doesn’t apply to your submission:

**Figure 2A-D.**

- Replicates: Each of these in vitro experiments were repeated independently for three times (batches) with technical triplicate each time.

- Refer to 'Figure 2. Legend (A-D)', Line 1023-1027

**Figure 2J-L.**

- Replicates: 6 total mice, 4 slides per mouse, from each experimental condition;

- Refer to 'Figure 2. legend (J-L)', Line 1032-1036

**Figure 3B.**

- Replicates: The *in vitro* experiments were repeated independently for three times (batches) with technical triplicate each time.

**Figure 3I-K.**

- Replicates: 6 total mice, 4 slides per mouse, from each experimental condition;

- Refer to 'Figure 3. legend (I-K)', Line 1059-1064

**Figure 4C-J.**

- Replicates: Analysis performed on 6 independent tumors for each experimental condition;

- Refer to ‘Figure 4. legend’, Line 1088-1090

**Figure 5A-B.**

- Replicates: Each of these in vitro experiments were repeated independently for three times (batches) with technical triplicate each time.

**Figure 5H-K, V.**

- Replicates: 6 total mice, 4 slides per mouse, from each experimental condition;

- Refer to 'Figure 5. legend (H-K, V)', Line 1105-1109

**Figure 6B, D.**

- Replicates: the experiments were repeated independently for three times (batches) with technical triplicate quantification each time.

**Figure 2-Supplement 2C-D.**

- Replicates: 6 total mice, 4 slides per mouse, from each experimental condition;

- Refer to 'Figure 2-S2. legend (C-D)', Line 1476-1477

**Figure 3-Supplement 1A.**

- Replicates: Each of these in vitro experiments were repeated independently for three times (batches) with technical triplicate each time.

**Figure 4-Supplement 1C-D.**

- Replicates: 6 different tumors for each condition, 4 slides per tumor;

- Refer to 'Figure 4-S1. legend (C-D)', Line 1511-1514

**Figure 4-Supplement 3A-D, G-J.**

- Replicates: 6 different tumors for each condition, 4 slides per tumor;

- Refer to 'Figure 4-S3. legend', Line 1548-1549

**Statistical reporting**

- Statistical analysis methods should be described and justified
- Raw data should be presented in figures whenever informative to do so (typically when N per group is less than 10)
- For each experiment, you should identify the statistical tests used, exact values of N, definitions of center, methods of multiple test correction, and dispersion and precision measures (e.g., mean, median, SD, SEM, confidence intervals; and, for the major substantive results, a measure of effect size (e.g., Pearson's r, Cohen's d)
- Report exact p-values wherever possible alongside the summary statistics and 95% confidence intervals. These should be reported for all key questions and not only when the p-value is less than 0.05.

Please outline where this information can be found within the submission (e.g., page numbers or figure legends), or explain why this information doesn’t apply to your submission:

(For large datasets, or papers with a very large number of statistical tests, you may upload a single table file with tests, Ns, etc., with reference to page numbers in the manuscript.)

**Figure 2E. Quantification of Soft agar assay**

Independent t-test

P=0.036

| Mean ± SEM of Control group | 1.000 ± 0.04027 N=9 |
| --- | --- |
| Mean ± SEM of Daam2-GOF | 1.329 ± 0.1076 N=9 |
| Difference between means | 0.3287 ± 0.1270 |
| 95% confidence interval | 0.02837 to 0.6290 |
| R squared | 0.4890 |

- Refer to 'Figure 2 Legend', Line 1027

**Figure 2I. Quantification of Bioluminescence**

Independent t-test

P=0.0079

| Mean ± SEM of Control group | 1.115 ± 0.2820 N=8 |
| --- | --- |
| Mean ± SEM of Daam2-GOF | 5.629 ± 1.513 N=7 |
| Difference between means | 4.514 ± 1.440 |
| 95% confidence interval | 1.403 to 7.624 |
| R squared | 0.4305 |

- Refer to 'Figure 2 Legend', Line 1032

**Figure 2L. Quantification of BrdU**

Independent t-test

P= 0.0019

| Mean ± SEM of Control group | 1.000 ± 0.1618 N=24 |
| --- | --- |
| Mean ± SEM of Daam2-GOF | 2.119 ± 0.2499 N=24 |
| Difference between means | 1.119 ± 0.3053 |
| 95% confidence interval | 0.4753 to 1.764 |
| R squared | 0.4416 |

- Refer to 'Figure 2 Legend’, Line 1035

**Figure 3E. Quantification of Bioluminescence**

Independent t-test

P=0.0325

| Mean ± SEM of Scram-Con | 0.9980 ± 0.3375 N=5 |
| --- | --- |
| Mean ± SEM of Daam2-shRNAi | 0.2957 ± 0.03760 N=7 |
| Difference between means | -0.7023 ± 0.2831 |
| 95% confidence interval | -1.333 to -0.07158 |
| R squared | 0.3810 |

- Refer to 'Figure 3. Legend', Line 1056

**Figure 3H. Quantification of Bioluminescence**

Independent t-test

p= 0.0049

| Mean ± SEM of Daam2+/- group | 1.000 ± 0.1858 N=9 |
| --- | --- |
| Mean ± SEM of Daam2-/- group | 0.3108 ± 0.07148 N=8 |
| Difference between means | -0.6892 ± 0.2089 |
| 95% confidence interval | -1.134 to -0.2439 |
| R squared | 0.4205 |

- Refer to 'Figure 3. Legend', Line 1059

**Figure 3K. Quantification of BrdU**

Independent t-test

P= 0.00001

| Mean ± SEM of Daam2+/- group | 1.000 ± 0.06979 N=24 |
| --- | --- |
| Mean ± SEM of Daam2-/- group | 0.4825 ± 0.05092 N=24 |
| Difference between means | -0.5175 ± 0.09161 |
| 95% confidence interval | -0.7035 to -0.3315 |
| R squared | 0.4769 |

Refer to 'Figure 2 Legend’, Line 1035

**Figure 5U. Quantification of Bioluminescence**

One-way analysis of variance (ANOVA) and followed by Tukey’s test for between-group comparisons.

* P=0.0001, ** P=0.0046, ***p=0.0016

| ANOVA summary |  |
| --- | --- |
| F | 15.02 |
| P value | < 0.0001 |
| R square | 0.5772 |
|  |  |
| * P value | 0.0001 |
| Mean ± SEM of Control | 1.000 ± 0.1382 N=12 |
| Mean ± SEM of Daam2 | 4.347 ± 0.7500 N=10 |
| Difference between means | 3.347 ± 0.6980 |
| 95% confidence interval | 1.891 to 4.803 |
| R squared | 0.5348 |
|  |  |
| ** P value | 0.0046 |
| Mean ± SEM of Daam2 | 4.347 ± 0.7500 N=10 |
| Mean ± SEM of Daam2+VHL | 1.020 ± 0.1510 N=6 |
| Difference between means | -3.327 ± 0.9886 |
| 95% confidence interval | -5.447 to -1.207 |
| R squared | 0.4472 |
|  |  |
| *** P value | 0.0016 |
| Mean ± SEM of VHL | 1.278 ± 0.2028 N=9 |
| Mean ± SEM of Daam2 | 4.347 ± 0.7500 N=10 |
| Difference between means | 3.069 ± 0.8157 |
| 95% confidence interval | 1.348 to 4.790 |
| R squared | 0.4544 |

- Refer to 'Figure 5. Legend', Line 1104-1105

**Figure 5V. Quantification of BrdU**

One-way analysis of variance (ANOVA) and followed by Tukey’s test for between-group

* P=0.0006, **P=0.0009, *** p<0.0001

| ANOVA summary |  |
| --- | --- |
| F | 10.06 |
| P value | < 0.0001 |
| R square | 0.3105 |

| * P value | 0.0006 |
| --- | --- |
| Mean ± SEM of Control | 0.9844 ± 0.1355 N=24 |
| Mean ± SEM of Daam2 | 1.572 ± 0.09040 N=24 |
| Difference between means | 0.5879 ± 0.1566 |
| 95% confidence interval | 0.2703 to 0.9055 |
| R squared | 0.2813 |
|  |  |
| ** P value | 0.0009 |
| Mean ± SEM of Daam2 | 1.572 ± 0.09040 N=24 |
| Mean ± SEM of Daam2+VHL | 1.101 ± 0.08487 N=24 |
| Difference between means | -0.4709 ± 0.1302 |
| 95% confidence interval | -0.7352 to -0.2067 |
| R squared | 0.2722 |
|  |  |
| *** P value | < 0.0001 |
| Mean ± SEM of VHL | 0.9256 ± 0.08036 N=24 |
| Mean ± SEM of Daam2 | 1.572 ± 0.09040 N=24 |
| Difference between means | 0.6467 ± 0.1236 |
| 95% confidence interval | 0.3964 to 0.8970 |
| R squared | 0.4186 |

- Refer to 'Figure 5. Legend', Line 1108

**Figure 6D. Quantification of degradation assay**

Half-life was determined via nonlinear, one-phase exponential decay analysis

P=0.0081

|  | VHL | VHL/Daam2 |
| --- | --- | --- |
| K | 0.1364 | 0.1884 |
| Half Life | 5.082 | 3.679 |
| Std. Error |  |  |
| K | 0.008151 | 0.01624 |
| 95% Confidence Intervals |  |  |
| K | 0.1182 to 0.1546 | 0.1522 to 0.2246 |
| Half Life | 4.485 to 5.862 | 3.086 to 4.553 |

- Refer to ‘Figure 6. legend’, Line 1132-1136

**Figure 2-S2E. Quantification of Bioluminescence**

Independent t-test

P=0.0019

| P value | 0.0019 |
| --- | --- |
| Mean ± SEM of Ctrl group | 1.001 ± 0.06756 N=20 |
| Mean ± SEM of D2 group | 1.433 ± 0.1169 N=17 |
| Difference between means | 0.4317 ± 0.1329 |
| 95% confidence interval | 0.1659 to 0.6976 |
| R squared | 0.1496 |

- Refer to ‘Figure 2-S2. Legend', Line 1475

**Figure 2-S2F. Quantification of BrdU**

Independent t-test

P<0.0001

| P value | < 0.0001 |
| --- | --- |
| Mean ± SEM of column A | 1.001 ± 0.05894 N=24 |
| Mean ± SEM of column B | 1.533 ± 0.05629 N=24 |
| Difference between means | 0.5324 ± 0.08236 |
| 95% confidence interval | 0.3652 to 0.6996 |
| R squared | 0.5442 |

- Refer to ‘Figure 2-S2. Legend', Line 1478

**Figure 4-S3E. Quantification of CD31**

Independent t-test

p=0.0331

| P value | 0.0331 |
| --- | --- |
| Mean ± SEM of Ctrl group | 1.000 ± 0.09996 N=24 |
| Mean ± SEM of D2-GOF | 1.498 ± 0.1618 N=24 |
| Difference between means | 0.4979 ± 0.2183 |
| 95% confidence interval | 0.04385 to 0.9519 |
| R squared | 0.1985 |

- Refer to ‘Figure 4-S3. Legend', Line 1547

**Figure 4-S3F. Quantification of CD31**

Independent t-test

P=0.0026

| P value | 0.0026 |
| --- | --- |
| Mean ± SEM of D2-Het | 0.9983 ± 0.07653 N=24 |
| Mean ± SEM of D2-KO | 0.6810 ± 0.04132 N=24 |
| Difference between means | -0.3173 ± 0.09217 |
| 95% confidence interval | -0.5096 to -0.1251 |
| R squared | 0.3721 |

- Refer to ‘Figure 4-S3. Legend', Line 1547

**Figure 4-S3K. Quantification of CD31**

One-way analysis of variance (ANOVA) and followed by Tukey’s test for between-group

* P=0.034, **P=0.044, *** p=0.0023

| ANOVA summary |  |
| --- | --- |
| F | 5.795 |
| P value | 0.0016 |
| R square | 0.2337 |
|  |  |
| * P value | 0.0348 |
| Mean ± SEM of Control | 1.001 ± 0.1003 N=24 |
| Mean ± SEM of Daam2 | 1.438 ± 0.1282 N=24 |
| Difference between means | 0.4372 ± 0.1959 |
| 95% confidence interval | 0.03383 to 0.8406 |
| R squared | 0.1662 |
|  |  |
| ** P value | 0.0445 |
| Mean ± SEM of Daam2 | 1.438 ± 0.1282 N=24 |
| Mean ± SEM of Daam2+VHL | 1.153 ± 0.06860 N=24 |
| Difference between means | -0.2849 ± 0.1372 |
| 95% confidence interval | -0.5623 to -0.007366 |
| R squared | 0.09954 |
|  |  |
| *** P value | 0.0023 |
| Mean ± SEM of VHL | 0.8582 ± 0.06529 N=24 |
| Mean ± SEM of Daam2 | 1.438 ± 0.1282 N=24 |
| Difference between means | 0.5802 ± 0.1727 |
| 95% confidence interval | 0.2258 to 0.9345 |
| R squared | 0.2947 |

- Refer to ‘Figure 4-S3. Legend', Line 1550

**Additional data files (“source data”)**

- We encourage you to upload relevant additional data files, such as numerical data that are represented as a graph in a figure, or as a summary table
- Where provided, these should be in the most useful format, and they can be uploaded as “Source data” files linked to a main figure or table
- Include model definition files including the full list of parameters used
- Include code used for data analysis (e.g., R, MatLab)
- Avoid stating that data files are “available upon request”

Please indicate the figures or tables for which source data files have been provided:
